# *Exocyst complex component 1* (*Exoc1)* loss in dormant oocyte disrupts c-KIT and growth differentiation factor (GDF9) subcellular localization and causes female infertility in mice

**DOI:** 10.1038/s41420-025-02291-5

**Published:** 2025-01-20

**Authors:** Chi Lieu Kim Nguyen, Yumeno Kuba, Hoai Thu Le, Hossam Hassan Shawki, Natsuki Mikami, Madoka Aoki, Nanako Yasuhara, Hayate Suzuki, Saori Mizuno-Iijima, Shinya Ayabe, Yuki Osawa, Tomoyuki Fujiyama, Tra Thi Huong Dinh, Miyuki Ishida, Yoko Daitoku, Yoko Tanimoto, Kazuya Murata, Woojin Kang, Masatsugu Ema, Yuji Hirao, Atsuo Ogura, Satoru Takahashi, Fumihiro Sugiyama, Seiya Mizuno

**Affiliations:** 1 Ph.D. Program in Human Biology, School of Integrative and Global Majors, University of Tsukuba, 1-1-1 Tennodai, Tsukuba, Ibaraki 305-8575 Japan; 2 Master’s Program in Medical Sciences, Graduate School of Comprehensive Human Sciences, University of Tsukuba, 1-1-1 Tennodai, Tsukuba, Ibaraki 305-8575 Japan; 3 Department of Comparative and Experimental Medicine, Nagoya City University Graduate School of Medical Sciences, Kawasumi, Mizuho-cho, Mizuho-ku, Nagoya 467-8601 Japan; 4 Research Fellow of the Japan Society for the Promotion of Science, Kojimachi Business Center Building, 5-3-1 Kojimachi, Chiyoda-ku, Tokyo 102-0083 Japan; 5 College of Medical Sciences, University of Tsukuba, 1-1-1 Tennodai, Tsukuba, Ibaraki 305-8575 Japan; 6 College of Biological Sciences, University of Tsukuba, 1-1-1 Tennodai, Tsukuba, Ibaraki 305-8572 Japan; 7 Laboratory Animal Resource Center in Trans-Border Medical Research Center, University of Tsukuba, 1-1-1 Tennodai, Tsukuba, Ibaraki 305-8575 Japan; 8 Experimental Animal Division, RIKEN BioResource Research Center, 3-1-1 Koyadai, Tsukuba, Ibaraki 305-0074 Japan; 9 International Institute for Integrative Sleep Medicine, University of Tsukuba, 1-1-1 Tennodai, Tsukuba, Ibaraki 305-8575 Japan; 10 Next Generation Human Disease Model Team, RIKEN BioResource Research Center, 3-1-1 Koyadai, Tsukuba, Ibaraki 305-0074 Japan; 11 Department of Stem Cells and Human Disease Models, Research Center for Animal Life Science, Shiga University of Medical Science, Seta Tsukinowa-cho, Otsu, Shiga 520-2192 Japan; 12 Division of Dairy Cattle Feeding and Breeding Research, Institute of Livestock and Grassland Science, National Agriculture and Food Research Organization, 2 Ikenodai, Tsukuba, Ibaraki 305-0901 Japan; 13 Bioresource Engineering Division, RIKEN BioResource Research Center, 3-1-1 Koyadai, Tsukuba, Ibaraki 305-0074 Japan

**Keywords:** Oogenesis, Exocytosis

## Abstract

A limited number of female germ cells support reproduction in many mammals. The follicle, composed of oocytes and supporting granulosa cells, forms the basis of oogenesis. Crosstalk between oocytes and granulosa cells is essential for the formation, dormancy, re-awakening, and maturation of oocytes. The oocyte expresses c-KIT and growth differentiation factor-9 (GDF-9), which are major factors in this crosstalk. The downstream signalling pathways of c-KIT and GDF-9 have been well-documented; however, their intra-oocyte trafficking pathway remains unclear. Our study reveals that the exocyst complex, a heterotetrameric protein complex important for tethering in vesicular transport, is important for proper intra-oocyte trafficking of c-KIT and GDF9 in mice. We found that depletion of oocyte-specific EXOC1, a component of the exocyst complex, impaired oocyte re-awakening and cyst breakdown, and inhibited granulosa cell proliferation during follicle growth. The c-KIT receptor is localised on the oocyte plasma membrane. The oocyte-specific *Kit* conditional knockout mice were reported to exhibit impaired oocyte re-awakening and reduced oocyte cyst breakdown. GDF9 is a protein secreted extracellularly in the oocyte. Previous studies have shown that *Gdf9* knockout mice impaired proliferation and granulosa cell multilayering in growing follicles. We found that both c-KIT and GDF9 abnormally stuck in the EXOC1-depleted oocyte cytoplasm. These abnormal phenotypes were also observed in oocytes depleted of exocyst complex members EXOC3 and EXOC7. These results clearly show that the exocyst complex is essential for proper intra-oocyte trafficking of c-KIT and GDF9. Inhibition of this complex causes complete loss of female fertility in mice. Our findings build a platform for research related to trafficking mechanisms of vital crosstalk factors for oogenesis.

## Introduction

In the mammalian ovary, the female reproductive lifespan is determined by the finite number of follicles [1]. Oocytes undergo various events in the ovary, including long dormancy periods from birth through sexual maturation and menopause, re-awakening from dormancy, maturation toward ovulation, and finally fertilisation with sperm in the oviduct. Granulosa cells are somatic cells that are always present in the immediate vicinity of the oocyte and are essential for the proper execution of these events in the ovary. In the prenatal and immediate postnatal mouse ovary, the oocytes are interconnected, termed as the oocyte cyst. Pre-granulosa cell invasion into this cyst causes cyst breakdown by postnatal day 5 (P5), and the oocytes become individualised single cells [2–4]. This event causes the construction of a follicle in which a single oocyte is surrounded by flattened monolayer granulosa cells. This dormant and smallest follicle located at the ovarian cortical region is the primordial follicle [5]. After sexual maturation, the oocytes in the primordial follicle re-awaken and the follicles grow dynamically. During follicle activation and growth, the granulosa cells surrounding the re-awakened oocyte become cuboidal and form the primary follicle. Subsequently, granulosa cells proliferate and multilayer, and the oocyte size increases cooperatively [6–8].

Studies using genetically modified mice revealed that the c-KIT receptor on the oocyte plasma membrane was essential for cyst breakdown and re-awakening as the stimuli receiver from granulosa cells [9, 10]. Factors downstream of c-KIT, such as FOXO3a and PTEN, have also been reported to contribute to re-awakening regulation [11, 12]. Growth differentiation factor 9 (GDF9), secreted from oocytes, is also essential for granulosa cell proliferation during follicle growth [13]. Therefore, c-KIT and GDF9 are the most important factors in the crosstalk; however, the intra-oocyte trafficking pathways of these molecules in oocytes are unknown.

Intracellular trafficking vesicles play important roles in protein and lipid transportation. The exocyst complex is a key component, functioning in vesicle tethering to specific sites on the plasma membrane [14]. This complex is a heterotetrameric protein complex, consisting of eight subunits, termed EXOC1–EXOC8 [15]. As male germ cell-specific *Exoc1*-conditional knockout (KO) mice have shown abnormal spermatogenesis [16], we hypothesised that the exocyst complex in oocytes could also be involved in oogenesis via regulating intra-oocyte trafficking of crosstalk factors. Such a function in oocytes could significantly enhance our understanding of the molecular dynamics underlying primordial follicle activation. Hence, this study aimed to determine the function of the exocyst complex and its involvement in intra-oocyte trafficking.

## Results

### *Exoc1* expression was confirmed in mouse oocytes

We tried to detect endogenous EXOC1 in wild-type mice using an anti-EXOC1 antibody and PA tagged-EXOC1 using *Exoc1*^*PA-N/PA-N*^ knock-in mice [16]; however, we failed to identify the signal. As immunostaining *Exoc1* in vivo was difficult, we used *Exoc1*^*+/LacZ*^ mice, in which the *LacZ* gene was inserted into the *Exoc1* locus to confirm *Exoc1* expression in oocytes [17]. X-gal staining showed signals in *Exoc1*^*+/LacZ*^ mice oocytes (Supplementary Fig. 1A). Exocyst complex gene expression was confirmed in oocytes using public RNA-seq data GSE143218 [18]. Most exocyst components were found to be expressed. Among *Exoc* genes, *Exoc1* was the most highly expressed in oocytes at all developmental stages (Supplementary Fig. 1B). *Exoc1* signal was found in oocyte and its expression in oocyte was also confirmed.

### *Gdf9*-Cre knock-in and oocyte-specific *Exoc1* conditional KO mice were generated

Novel B6-*Gdf9*^*em1(Cre)Utr*^ (hereafter: *Gdf9*^*Cre*^) mice were generated (Supplementary Fig. 2) for producing oocyte-specific *Exoc1* deletion mice. Mating with this novel Cre driver and *Gt(ROSA)26Sor*^*tm1(CAG-EGFP/tDsRed)Utr*^ (hereafter: *ROSA*^*GRR*^) [19] showed Cre recombination only in oocytes at P3 (Supplementary Fig. 3), but not in male germ cells (Supplementary Fig. 4). A minor recombination was found in testis and cerebellum (Supplementary Fig. 5). Similar to the *Gdf9* KO mice phenotype [20], enlarged oocytes were found in primary follicles; no follicles grew further than secondary follicles in the *Gdf9*^*Cre/Cre*^ ovary (Supplementary Fig. 6). This abnormal phenotype was thought to have occurred because of GDF9 protein dysfunction caused by 2A sequence addition [21]. As these abnormalities were absent in *Gdf9*^*+/Cre*^ mice, we decided to use *Exoc1*^*flox/flox*^*::Gdf9*^*+/Cre*^ as cKO mice for further study.

### Follicle growth was disrupted in oocyte-specific *Exoc1* cKO mice

**Fig. 1:**
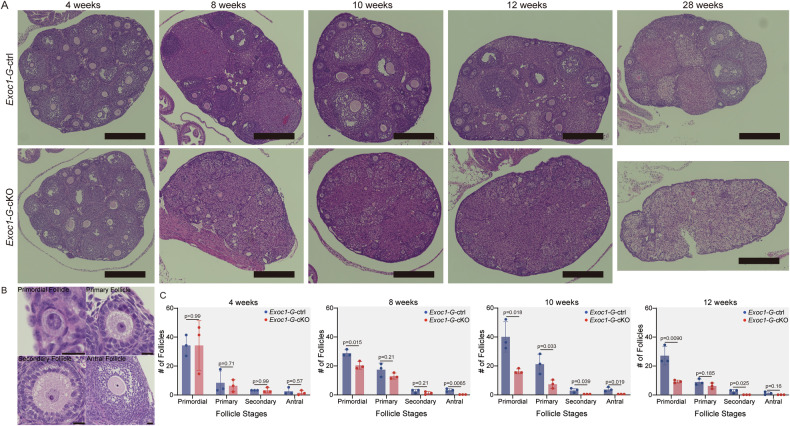
Follicle growth in *Exoc1-G-*cKO mice. Follicle growth in the ovaries of 4, 8, 10, and 12-week-old *Exoc1-G*-cKO (*Exoc1*^*flox/flox*^::*Gdf9*^*+/Cre*^) and control *Exoc1-G*-ctrl (*Exoc1*^*+/flox*^::*Gdf9*^*+/Cre*^) mice. **A** Representative Haematoxylin and Eosin staining images of *Exoc1-G*-cKO mice ovaries. No antral follicles were observed in *Exoc1-G*-cKO mice ovaries that were eight weeks old. No follicles were observed at any stage in 28-week-old *Exoc1-G*-cKO mice. Scale bar = 500 μm. **B** Representative Haematoxylin and Eosin staining images of primordial, primary, secondary and antral follicle in 4-week-old *Exoc1-G*-cKO ovary. Scale bar = 20 μm. **C** Follicle count in ovaries in *Exoc1-G*-cKO mice. No secondary follicles were observed in the *Exoc1-G*-cKO mice that were 10 and 12 weeks old. n = 3, Student’s *t* test.

To investigate *Exoc1* functions in oocytes, ovaries of 4, 8, 10, and 12-week-old *Exoc1*^*flox/flox*^*::Gdf9*^*+/Cre*^ (hereafter: *Exoc1-G-*cKO) mice were collected. Secondary and antral follicles were found in *Exoc1*^*flox/+::*^*Gdf9*^*+/Cre*^ (hereafter: *Exoc1-G-*ctrl) mice ovaries of all ages, but not in the eight-week-old *Exoc1-G-*cKO mice (Fig. 1). The number of primary follicles tended to decrease in *Exoc1-G-*cKO mice from 10 weeks old (Fig. 1).

EXOC1 not only contributes to tethering as a exocyst complex member but is also involved in SNARE complex construction [22]. To determine the cause of abnormal follicular growth exhibited by *Exoc1*-*G*-cKO mice being exocyst complex dysfunction, we generated *Exoc3*^*flox/flox*^*::Gdf9*^*+/Cre*^ and *Exoc7*^*flox/flox*^*::Gdf9*^*+/Cre*^ (hereafter: *Exoc3-G-*cKO and *Exoc7-G-*cKO, respectively) mice. *Exoc3* and *Exoc7* involvement in SNARE complex formation has not been reported; their expression levels in mice oocytes were second and third compared to *Exoc1* (Supplementary Fig. 1). Consistency was found in *Exoc3-G-*cKO, *Exoc7-G-*cKO, and *Exoc1-G-*cKO, which illustrated no growing follicle in the adult stages (Supplementary Fig. 7). These findings suggest that the exocyst protein complex is important for folliculogenesis in mice.

The number of oocytes in primordial and primary follicles were significantly reduced in eight-week-old and older *Exoc1-G-*cKO mice (Fig. 1). The immunofluorescence for MVH, a germ cell marker, was absent in 28-week-old *Exoc1-G-*cKO mice oocytes (Supplementary Fig. 8), which suggests the essential roles of EXOC1 functioning along with other subunits in maintaining the female reproductive capacity. Collectively, *Exoc1* was required for the follicle growth.

### Follicle growth failure was observed in the *Exoc1* cKO ovary under normal endocrine conditions

**Fig. 2:**
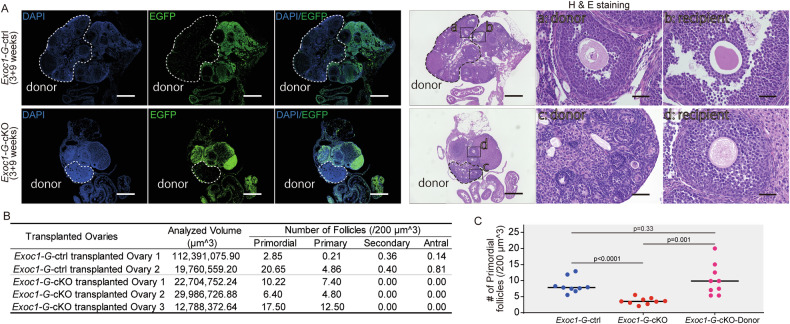
Ovarian transplantation of *Exoc1-G*-cKO ovaries into *ROSA*^*GRR/GRR*^ ovaries. Images of harvested ovaries nine weeks after ovary transplantation from three-week-old *Exoc1-G*-cKO (*Exoc1*^*flox/flox*^::*Gdf9*^*+/Cre*^) and *Exoc1-G*-ctrl (*Exoc1*^*+/flox*^::*Gdf9*^*+/Cre*^) mice into three-week-old *ROSA*^*GRR/GRR*^ mice. **A** Representative immunofluorescence images for green fluorescence protein and Haematoxylin and Eosin staining. Dashed circles: transplanted donor ovarian regions a, c: Oocytes in donor ovaries. b, d: Oocytes in recipient ovaries. Scale bar = 500 µm (at low magnification), scale bar = 50 µm (high magnification). **B** Follicle growth in donor ovaries. Primordial and primary follicles, but not secondary follicles, were found in the *Exoc1-G*-cKO donor ovaries. **C** Comparison of transplanted and non-transplanted *Exoc1-G*-cKO mice ovaries. The number of primordial oocytes was significantly higher in transplanted *Exoc1-G*-cKO ovaries than in non-transplanted *Exoc1-G*-cKO mice ovaries (9 + 3 and 12 weeks). Three ovarian regions per mouse were measured, and the number of primordial oocytes in nine regions, 3 regions ×3 mice, was plotted. One-way analysis of variance.

The absence of EXOC1 in oocytes caused follicle growth failure and premature depletion of primordial follicles (Fig. 1). Reproductive endocrine hormones secreted by the hypothalamus and pituitary gland regulate follicle growth and ovulation [23]. Cre recombination was confirmed in the cerebellum of *Gdf9-Cre* mice (Supplementary Fig. 5C), and the exocyst complex was implicated in proper brain morphogenesis and receptor orientation at synapses [24, 25]. We sought to verify the oocyte abnormal phenotypes in *Exoc1-G-*cKO occurring because of a disturbance in the reproductive endocrine system. We transplanted three-week-old *Exoc1-G-*cKO mouse ovaries into recipient *ROSA*^*GRR/GRR*^ mice ovaries and harvested them nine weeks later. We examined donor (*Exoc1-G-*cKO) oocytes in the ovary regions using green fluorescence protein immunofluorescence and found no growing follicles (Fig. 2A, B). In contrast, wildtype ovaries transplanted into *ROSA*^*GRR/GRR*^ mice were capable of ovulation. Donor ovary oocytes developed into 2-cell embryos via in vitro fertilisation (Supplementary Fig. 9). This result indicated that EXOC1 deficiency in the oocyte caused the follicle growth defects.

The number of primordial follicles significantly increased in transplanted *Exoc1-G-*cKO ovaries compared to those in non-translated *Exoc1-G-*cKO ovaries. The number between transplanted *Exoc1-G-*cKO and *Exoc1-G-*ctrl ovaries showed no significant differences (Fig. 2C). This result suggested that the early depletion of the primordial follicle pool observed in *Exoc1-G-*cKO mice was not directly caused by EXOC1 deficiency in oocyte itself.

### Oocyte re-awakening was impaired in *Exoc1* cKO and subcellular c-KIT localisation was improper

**Fig. 3:**
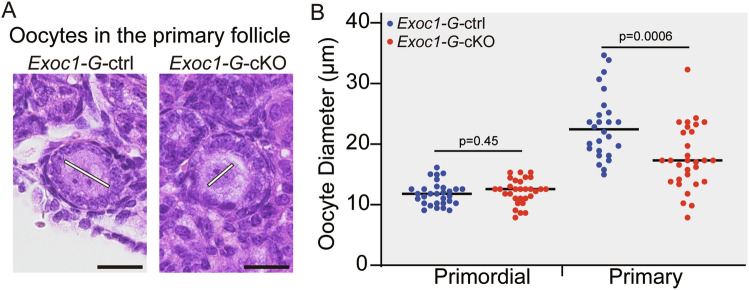
Diameter of the oocytes in primary follicles in *Exoc1-G*-cKO mice. **A** Morphology of oocytes in primary follicles in *Exoc1-G*-cKO (*Exoc1*^*flox/flox*^::*Gdf9*^*+/Cre*^) ovaries compared to that of the control (*Exoc1*^*+/flox*^::*Gdf9*^*+/Cre*^). White line: measured diameter. Scale bar = 20 μm. **B** Diameter of oocytes in primordial and primary follicles in *Exoc1-G*-cKO mice. The diameter of oocytes in primordial follicles between control and *Exoc1-G*-cKO mice showed no significant differences. In contrast, the oocytes in primary follicles in *Exoc1-G*-cKO mice showed significantly shorter diameters than those in control mice. n = 3, Student’s *t* test.

EXOC1 deletion in oocytes caused follicle growth failure (Fig. 2). To confirm the oocyte growth states in primordial and primary follicles, we measured their diameters in 10-week-old *Exoc1-G-*cKO mice with significantly impaired follicular growth (Fig. 1). The diameter of *Exoc1-G-*cKO mice oocytes in primary follicles was significantly smaller than that of *Exoc1-G-*ctrl mice, whereas those of oocytes in primordial follicles did not differ (Fig. 3). This finding implies that the re-awakening process was disrupted in *Exoc1-G-*cKO oocytes.

**Fig. 4:**
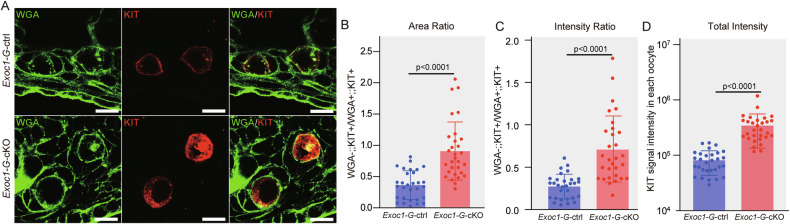
Impairment of c-KIT trafficking in *Exoc1-G*-cKO mice. **A** Representative immunofluorescence images of 10-week-old *Exoc1-G*-cKO (*Exoc1*^*flox/flox*^::*Gdf9*^*+/Cre*^) and *Exoc1-G*-ctrl (*Exoc1*^*+/flox*^::*Gdf9*^*+/Cre*^) mice ovaries. The c-KIT signals were observed mainly on the plasma membrane in oocytes in primary follicles of control mice. In contrast, extensive c-KIT dot-like signals were detected in the oocyte cytoplasm in primary follicles of *Exoc1-G*-cKO mice. Scale bar = 20 μm. **B** Area ratio of plasma membrane c-KIT (c-KIT co-localised with wheat germ agglutinin (WGA), WGA+::KIT+) to cytoplasmic c-KIT (c-KIT not co-localised with WGA, WGA-::KIT+). n = 3, Student’s *t* test. **C** Signal intensity ratio of plasma membrane c-KIT to cytoplasmic c-KIT. n = 3, Student’s *t* test. **D** Intensity of the total KIT signal in each oocyte. n = 3, Student’s *t* test.

*Exoc1-G-*cKO mice exhibit a failure in oocyte re-awakening and premature depletion of primordial oocytes, similar to oocyte-specific *Kit* cKO mice [10]. The *Kit* gene encodes c-KIT, a transmembrane receptor on the oocyte membrane that is required for dormant oocyte re-awakening [10]. Dot-like c-KIT signals were found in the oocyte cytoplasm in *Exoc1-G-*cKO primary follicles, whereas c-KIT signals were mainly located in oocyte plasma membrane of *Exoc1-G-*ctrl (Fig. 4A). Measuring the area ratio of plasma membrane c-KIT (colocalised with wheat germ agglutinin, WGA) to cytoplasmic c-KIT (not colocalised with WGA) revealed that *Exoc1-G-*cKO oocytes had a higher proportion of cytoplasmic c-KIT than *Exoc1-G-*ctrl mouse oocytes (Fig. 4B). Comparing signal intensity ratios showed that c-KIT signals were significantly stronger in the *Exoc1-G-*cKO oocyte cytoplasm in primary follicles (Fig. 4C). Total c-KIT signal intensity in oocytes was significantly higher in *Exoc1-G-*cKO mice (Fig. 4D). Stem cell factor (SCF) binding to c-KIT causes dimerisation, autophosphorylation, internalisation, and degradation of c-KIT [26, 27]. When activated and internalised c-KIT degradation is impaired, downstream signalling increases instead of decreasing [28]. *Kit* cKO female mice failed to show primordial follicle activation [10]. In EXOC1-deficient oocytes, trafficking of the newly translated c-KIT to the plasma membrane could be disrupted, rather than the degradation of internalised active c-KIT.

### Artificial activation of PI3K-Akt rescued oocyte re-awakening failure in *Exoc1* cKO

**Fig. 5:**
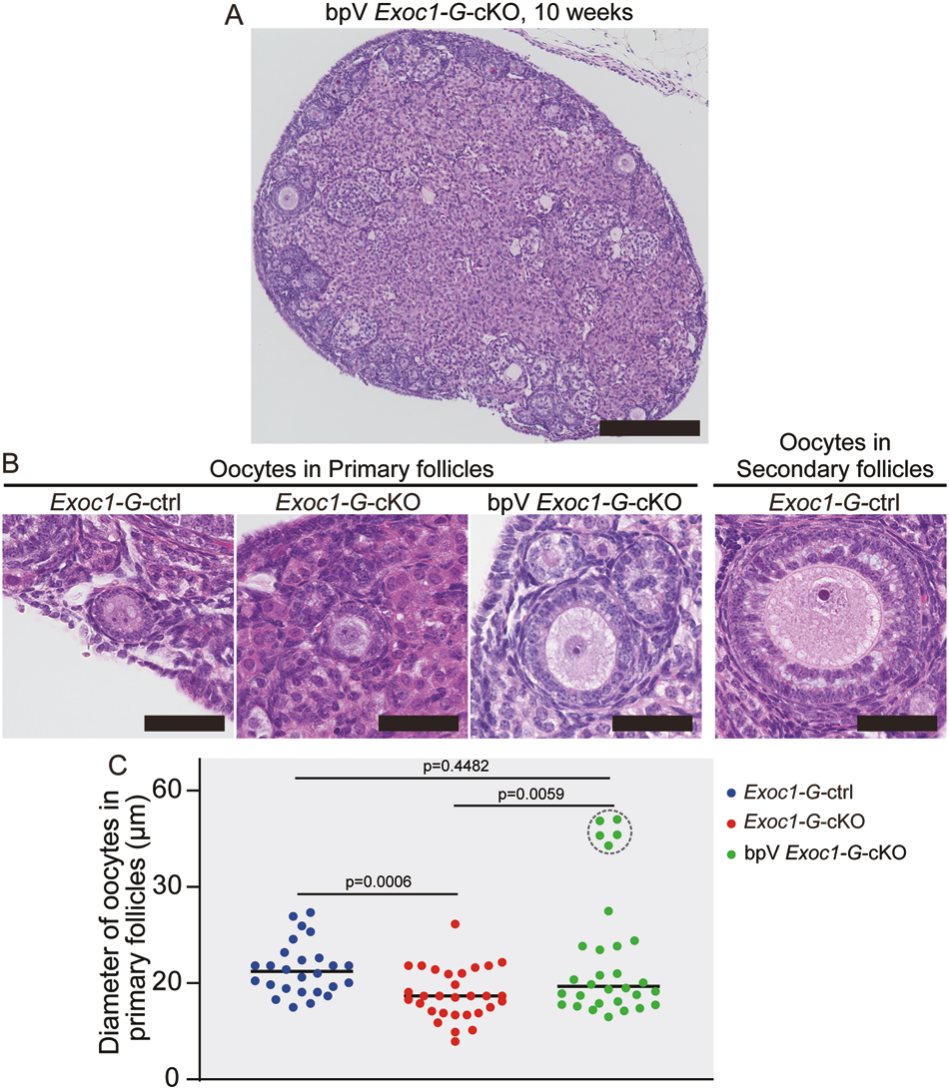
Rescue of oocyte re-awakening failure in *Exoc1-G*-cKO mice using a PTEN inhibitor. **A** Macroscopic Haematoxylin and Eosin-stained images of PTEN inhibitor bpV-treated *Exoc1-G*-cKO (bpV-*Exoc1-G*-cKO) mice ovaries. Scale bar = 500 μm. **B** Representative primary follicles of each mouse genotype (10 weeks old). The control mice were *Exoc1*^*+/flox*^::*Gdf9*^*+/Cre*^, referred to as *Exoc1-G-*ctrl. Scale bar = 50 μm. **C** Plots of the oocyte sizes in primary follicles of each mouse genotype at 10 weeks of age. n = 3, one-way analysis of variance. Oocyte diameters in primary follicles in bpV *Exoc1-G*-cKO mice were significantly longer than those in *Exoc1*-*G*-cKO mice. In the bpV *Exoc1-G*-cKO group, enlarged oocytes appeared in primary follicles (plots circled by dashed line), which were absent in the control group.

We hypothesised that the oocyte re-awakening failure in *Exoc1-G-*cKO was caused by c-KIT dysfunction. To verify this, we performed rescue experiments on the c-KIT downstream pathway. In mice oocytes, c-KIT regulates the PI3K/Akt/FOXO3 pathway [10]. PTEN suppresses oocyte re-awakening via inhibiting Akt [7]. A previous report showed that injecting bisperoxovanadium (bpV), a PTEN inhibitor, increased the mice litter size [29]. Therefore, we attempted to rescue the c-KIT downstream pathway using a bpV injection. BpV-treated *Exoc1-G-*cKO mice did not have growing follicles in their ovaries (Fig. 5A). In primary follicles, bpV-treated *Exoc1-G-*cKO oocytes were significantly larger than non-treated *Exoc1-G-*cKO oocytes (Fig. 5B, C). Some bpV-treated *Exoc1-G-*cKO oocytes in primary follicles were as large as control mice oocytes in secondary follicles (Fig. 5B). Wildtype female mice treated with bpV did not exhibit oversized oocytes in the primary follicles and showed a reduced number of primary follicles (Supplementary Fig. 10). These results suggested that the oocyte re-awakening failure in *Exoc1* cKO mice was caused by the impaired c-KIT and its downstream signalling.

### GDF9 secretion was impaired in *Exoc1* cKO mice

In bpV-treated *Exoc1-G-*cKO mice, the oocyte size in primary follicles increased, but the granulosa cell multilayering that should have accompanied this change did not occur (Fig. 5B). PTEN inhibition partially rescued the oocyte re-awakening failure but not follicle growth impairment in *Exoc1-G-*cKO mice. To enable coordinated and appropriate follicular growth, oocytes and granulosa cells exchange crosstalk factors via paracrine signalling [30]. We hypothesised that the oocyte-derived secretion that promoted granulosa cell proliferation was disrupted. GDF9 is important for follicle growth. GDF9 secreted by oocytes promotes the granulosa cells around them to proliferate [31]. Oocytes in primary follicles grow excessively in *Gdf9* KO mice, but the surrounding granulosa cells do not multilayer, and follicle growth stops at this stage [20]. Hence, we focused on GDF9 localisation in oocytes.

**Fig. 6:**
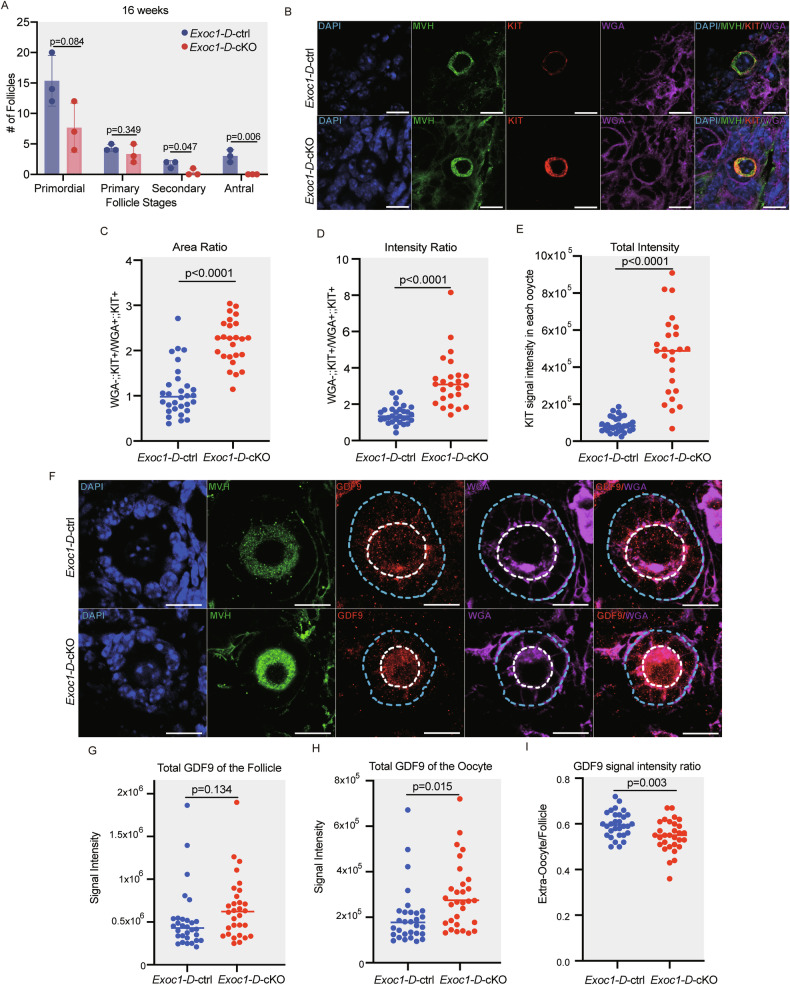
*Exoc1* deletion during adult stage impairs the subcellular location of growth differentiation factor-9 (GDF9). Tamoxifen was injected into eight-week-old *Exoc1*-*D*-cKO (*Exoc1*^*flox/flox*^::*Ddx4*^*+/CreERT2*^) and *Exoc1*-*D*-ctrl (*Exoc1*^*flox/+*^::*Ddx4*^*+/CreERT2*^) mice. **A** Oocyte count in ovaries in 16-week-old mice. No growing follicles were observed in the *Exoc1*-*D*-cKO mice. N = 3, Student’s *t* test. **B** Representative immunofluorescence images of 16-week-old *Exoc1*-*D*-cKO and control mice ovaries. The c-KIT signals were observed mainly on the plasma membrane of oocytes in the primary follicles of control mice, whereas exclusive c-KIT signals were observed in the cytoplasm of oocytes in primary follicles of *Exoc1*-*D*-cKO mice. Scale bar = 20 μm **C** Area ratio of plasma membrane c-KIT (c-KIT co-localised with wheat germ agglutinin (WGA), WGA + ::KIT + ) to cytoplasmic c-KIT (c-KIT not co-localised with WGA, WGA-::KIT+). **D** Intensity ratio of plasma membrane to cytoplasmic c-KIT. **E** Intensity of the total KIT signal in each oocyte. N = 3, Student’s *t* test. **F** Representative immunofluorescence images of 16-week-old *Exoc1*-*D*-cKO and control mice ovaries. Most GDF9 signals were found in the extra-oocyte region of the *Exoc1*-*D*-ctrl mice primary follicles. However, GDF9 signals were mostly localised in ooplasm of the primary follicles of *Exoc1*-*D*-cKO mice. Blue dashes: Follicle area. White dashes: oocyte areas in primary follicles. Scale bar = 20 μm **G** Plot of the sum of the GDF9 signal intensities in each follicle. No significant differences were observed among the two groups. **H** Plot of the GDF9 signal intensity in the oocytes in primary follicles only. Oocyte GDF9 levels in *Exoc1*-*D*-cKO mice were significantly higher than those in the control. **I** The ratio of GDF9 signal intensity between the extra-oocyte and follicle area. GDF9 signals were found mainly in oocyte cytoplasm of *Exoc1*-*D*-cKO primary follicles. N = 3, Student’s *t* test.

The GDF9 protein showed abnormal behaviour in *Gdf9*^*Cre/Cre*^ (Supplementary Fig. 6). Therefore, to precisely understand GDF9 localisation in oocyte-depleted EXOC1, we employed the B6-*Ddx4*^*em1(CreERT2)Utr*^ (hereafter: *Ddx4*^*+/CreERT2*^) mouse [21] as a Cre driver. This *CreERT2* knock-in strain, both heterozygous and homozygous, showed normal oogenesis. Tamoxifen administration to eight-week-old mice showed 100% Cre recombination efficiency in germ cells [21]. We injected tamoxifen to eight-week old *Exoc1*^*flox/flox*^*::Ddx4*^*+/CreERT2*^ (hereafter: *Exoc1-D-*cKO) and *Exoc1*^*flox/flox*^*::Ddx4*^*+/+*^ (hereafter: *Exoc1-D*-ctrl) mice, and collected 16-week-old ovaries. *Exoc1-D-*cKO was consistent with *Exoc1-G-*cKO, in which no growing follicles and c-KIT abnormal location were found (Fig. 6A–E), emphasizing that the anomalies found in *Exoc1-G-*cKO were caused by EXOC1 depletion in oocytes. We then investigated GDF9 localisation. Abundant GDF9 signals were found in the extra-oocyte region of *Exoc1-D*-ctrl primary follicles, whereas GDF9 signals were mainly found in the oocyte cytoplasm of *Exoc1-D-*cKO primary follicles (Fig. 6F). A quantitative analysis of the total GDF9 signal in the entire follicle revealed no significant differences between the *Exoc1-D*-cKO and *Exoc1-D-*ctrl mice follicles (Fig. 6G). The signal intensity in the *Exoc1-D-*cKO primary follicle oocyte was higher than that of the control group (Fig. 6H). GDF9 signal intensity was significantly lower in the extra-oocyte region of *Exoc1-D-*cKO primary follicles compared to controls (Fig. 6I). Therefore, EXOC1 depletion interfered with GDF9 secretion from the oocyte to granulosa cells.

### Improper localisation of c-KIT and GDF9 interfered with their downstream pathways

**Fig. 7:**
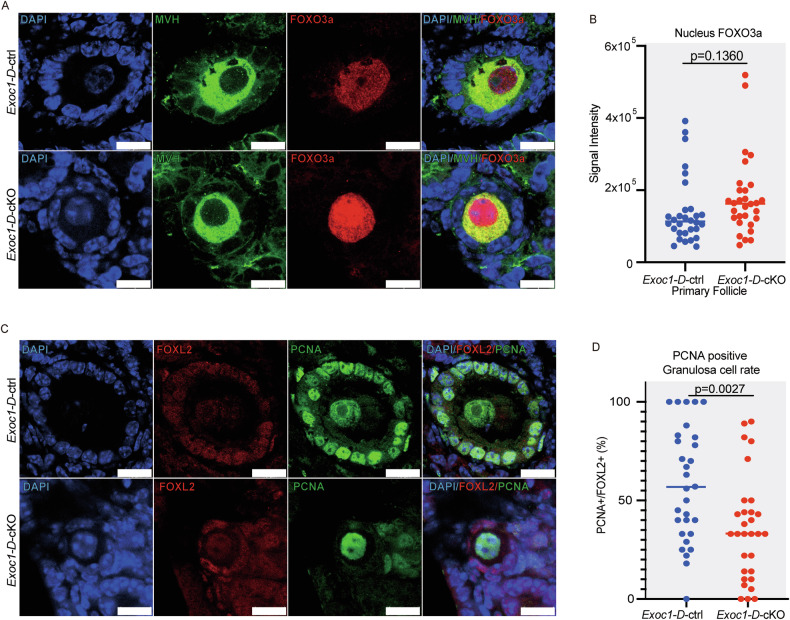
Downstream pathways of c-KIT and growth differentiation factor-9 (GDF9). **A** Representative immunofluorescence images of 16-week-old *Exoc1*-*D-*cKO (*Exoc1*^*flox/flox*^::*Ddx4*^*+/CreERT2*^) and *Exoc1*-*D-*ctrl (*Exoc1*^*flox/+*^::*Ddx4*^*+/CreERT2*^) primary follicles. FOXO3a signals concentrated in the nucleus of *Exoc1*-*D-*cKO oocytes in primary follicles. Scale bar = 10 µm. **B** The signal intensity of FOXO3a in the nucleus of oocytes in primary follicles. n = 3, Student’s *t* test. **C** Representative PCNA and FOXL2, a marker of granulosa cells, co-immunofluorescence images of 16-week-old *Exoc1*-*D-*cKO and control primary follicles. Scale bar = 10 µm. **D** The percentage of PCNA-positive granulosa cells in the primary follicles of *Exoc1-D-*cKO mice was significantly lower than that in the control. n = 3, Student’s *t* test.

*Exoc1* deletion impaired c-KIT localisation (Fig. 4) and GDF9 (Fig. 6). We investigated the c-KIT and GDF9 downstream pathways. Activation of c-KIT caused FOXO3a translocation from the oocyte nucleus to cytoplasm [32], promoting follicle activation. FOXO3a immunostaining showed that signals of this protein were more concentrated in the *Exoc1-D-*cKO oocyte nucleus than in that of *Exoc1-D-*ctrl oocyte although not with a statically significant difference (Fig. 7A, B). Hence, deleting EXOC1 in oocytes partially disrupted c-KIT-induced primordial follicle activation pathways. Conversely, GDF9 promotes granulosa cell proliferation in the surrounding area [31]. PCNA immunofluorescence revealed significantly fewer PCNA-positive granulosa cells in *Exoc1-D-*cKO primary follicles (Fig. 7C, D). This finding describes a defect in the downstream pathway of GDF9 secretion from oocytes. Collectively, the depletion of EXOC1 in oocytes disrupted the transport of c-KIT and GDF9, which negatively affected their downstream pathways.

### *Exoc1* deletion before birth interrupted cyst breakdown and folliculogenesis

**Fig. 8:**
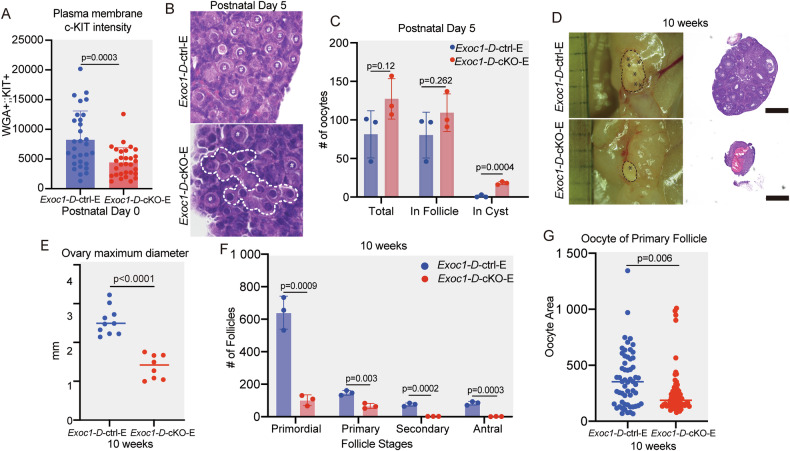
*Exoc1* deletion during foetal ovarian development significantly disrupts folliculogenesis. **A** Intensity level of plasma membrane c-KIT. The c-KIT located in plasma membrane of *Exoc1-D-*cKO-E (*Exoc1*^*flox/flox*^::*Ddx4*^*+/CreERT2*^) oocytes at postnatal day 0 (P0) compared to control group *Exoc1*-*D*-ctrl-E (*Exoc1*^*flox/+*^::*Ddx4*^*+/CreERT2*^. **B** Representative Haematoxylin and Eosin staining images of *Exoc1-D-*cKO-E ovaries at P5. Dashed white line: oocytes in the cyst. Hashtag: single follicles. **C** Total number of oocytes in ovaries, follicles, and cysts of both groups. The number of oocytes in cyst of *Exoc1-D-*cKO-E ovaries was significantly higher than those in control. n = 3, Student’s *t* test. **D** Representative ovaries and Macroscopic Haematoxylin and Eosin-stained images of *Exoc1-D*-cKO-E and *Exoc1-D-*ctrl-E mice that were 10 weeks old. Dashed black line: ovary area. Asterisk: follicles observed on the ovary surface. Scale bar = 500 µm. **E** Ovary maximum diameter. *Exoc1-D*-cKO-E ovaries were smaller than those of the control group. **F** Oocyte counts in whole ovaries at 10 weeks of age. Secondary and antral follicles were absent in the *Exoc1-D*-cKO-E mice. n = 3, Student’s *t* test. **G** Oocyte area of primary follicles. Oocytes in primary follicles of *Exoc1-D*-cKO-E mice were significantly smaller than those in control group. n = 3, Student’s *t* test.

Cyst breakdown is important for oocyte individualisation and follicle formation [3, 4]. *Exoc1* is essential for regulating cytoplasmic division in mice spermatocytes [16], and c-KIT facilitates cyst breakdown [9]. Cyst breakdown mainly occurs prenatally; therefore, it could not be evaluated with *Exoc1-G-*cKO and *Exoc1-D-*cKO mice. To investigate the effect of *Exoc1* deletion during foetal ovarian affect cyst breakdown, we induced Cre recombination in *Exoc1*^*flox/flox*^*::Ddx4*^*+/CreERT2*^ mice at E15 and named these mice *Exoc1-D-*cKO-*E*. We tested oral tamoxifen administration methods [33] with *Ddx4*^*+/CreERT2*^*::ROSA*^*GRR/+*^ mice and found 100% recombination in oocytes at P0 (Supplementary Fig. 11). Following oral tamoxifen administration, oocyte-specific *Exoc1* deletion mice from the embryonic stage were generated. The plasma membrane c-KIT signal was significantly lower in *Exoc1-D-*cKO-*E* than in control mice at P0 (Fig. 8A). We then examined P5 when cyst breakdown was supposed to be completed. On P5, cysts were found in *Exoc1-D-*cKO-*E* (Fig. 8B, C), indicating EXOC1 requirement for this event. At the adult stage, ovary size, follicle number, and oocyte diameter in *Exoc1-D-*cKO-*E* mice significantly reduced, showing the same phenotype as *Kit* cKO (Fig. 8D–G). These results suggested that EXOC1 deletion during foetal development disrupts cyst breakdown via regulating c-KIT and considerably impaired folliculogenesis.

## Discussion

In this study, we demonstrated that EXOC1 in the mouse oocyte was essential for the intra-oocyte transport of c-KIT and GDF9, the most important crosstalk factors between the oocyte and granulosa cells. Our findings highlight the critical role of *Exoc1* in maintaining the proper trafficking process of crosstalk factors required during folliculogenesis.

Although *Exoc1* deletion could have caused abnormal c-KIT localisation, FOXO3 nuclear localisation showed no significant differences (Fig. 8). The c-KIT signalling pathway is not the only reported mechanism that induces FOXO3a translocation. *Lhx8* controls primordial follicle activation and postnatal folliculogenesis [34]. ELAVL2-directed RNA networks are implicated in maintaining primordial follicle quiescence, suggesting a complex interplay of molecular signals in follicle dormancy and activation [35]. Environmental factors such as hypoxia or organelle activity such as mitochondria also play regulatory roles in this event [36, 37]. The existence of c-Kit rescue pathways and their role in follicle activation, however, remains unexplored and requires further investigation.

This study has not revealed that which type of vesicle is related to c-KIT endosomal trafficking. ARF6 and the transferrin receptor (TfR), two key markers of recycling endosomes, exhibit c-KIT partial co-localisation [38], suggesting that EXOC1 could regulate ARF6 and TfR-positive recycling endosomes in c-KIT vesicular trafficking. This hypothesis is supported by observations in Kasumi-1 cells, which are from a human acute myeloid leukaemia cell line, wherein mutant c-KIT accumulates in TfR-positive recycling endosomes [39]. Our study found a predominantly high c-KIT cytoplasmic signal (Fig. 4A), with some existence noted in the oocyte membrane region. This pattern implies the existence of *Exoc1*-independent trafficking pathway(s) of c-KIT transport, which could have partially reduced FOXO3a signals in *Exoc1-D-*cKO (Fig. 8A, B). These pathway(s) may either compensate for or operate concurrently with *Exoc1*-dependent mechanisms, which our study has not extensively explored.

GDF9 signals were abundantly observed in the oocyte regions; however, EXOC1 association with this factor secretion remains unexplored. *Ggpps* deletion in oocytes caused excessive oocyte growth without corresponding granulosa cell proliferation [40]. This phenomenon was similar to the phenotype observed in *Gdf9* KO mice, suggesting similar developmental disruptions. *Ggps1*e synthesises geranylgeranyl diphosphate and plays a crucial role in modifying the C-terminus of RAB small GTPase proteins, which are essential components in intracellular trafficking processes. Geranylgeranyl diphosphate depletion in the oocyte impaired RAB27 geranylgeranylation and reduced CDC42 activity, which disrupted GDF9 secretion [40]. Further emphasizing the importance of trafficking processes, the interaction between CDC42 and RAB27 with the exocyst complex was proven [41, 42]. These findings imply the interaction between EXOC1 and small G-proteins in GDF9 secretion. Oocyte microvilli-derived vesicles were correlated with GDF9 secretion [43]. This relationship between microvilli structure and a key crosstalk factor suggests a complex interplay in follicular development. Therefore, EXOC1 involvement in these networks could be integral to the coordination of intra-oocyte trafficking and signalling processes that are critical for proper ovarian function.

*Exoc1-D-*cKO-*E* models showed impaired cyst breakdown and considerable ovary shrinkage, which were absent in *Exoc1-G-*cKO nor *Exoc1-D-*cKO. The ovary shrinkage was similar to that observed in *Kit* cKO (*Kit*^*flox/-*^*:: Ddx4-cre*^*1Dcas/J*^) models [10]. The number of follicles dropped significantly, which was not shown in the *Kit* cKO, suggesting that *Exoc1* could also have functions that contributed to oocyte viability during cyst breakdown that were absent in *Kit* cKO. JAG1, a oocyte secreting protein, promotes cyst breakdown via activating Notch signalling in granulosa cells [44]. The possibility of *Exoc1* involvement in the intra-oocyte trafficking of these oocyte secretion factors require examination in the future. Germ cells are encapsulated by two sources of pre-granulosa cells, leading to two distinct waves of folliculogenesis [45, 46]. Disruption of one or both waves could diminish folliculogenesis. However, *Exoc1* function in the growth of the two waves has not been discovered in this study.

We discovered that EXOC1 was necessary for crosstalk factor intra-oocyte trafficking, which was in turn required for oocyte re-awakening, follicle growth, and cyst breakdown. With the identification of several intracellular vesicle trafficking-related factors that collaborate with the exocyst complex, this study provides a foundation for identifying factors important for folliculogenesis occurrence. Our findings have suggested the importance of not only *Exoc1, Exoc3* and *Exoc7* but also exocyst complex in female germ cell development. Oocyte-specific *Exoc5* cKO mice experienced the similar apprehension in follicle growth [47], which supports our argument regarding the importance of the exocyst complex in folliculogenesis. Our research provided valuable insights into the roles of *Exoc1* in the crosstalk trafficking process of c-KIT and GDF9, which could be the explanation for the arrested follicle growth in *Exoc5* cKO mice reported previously. Further investigations are necessary to understand the roles in the trafficking process of other exocyst complex members.

## Methods and materials

### Mice

Mice were maintained in plastic cages under specific pathogen-free conditions at 23.5 °C ± 2.5 °C and 52.5% ± 12.5% relative humidity under a 14-h light/10-h dark cycle at the Laboratory Animal Resource Center at the University of Tsukuba. The mice had free access to commercial chow (MF diet; Oriental Yeast Co. Ltd., Tokyo, Japan) and filtered water. ICR and C57BL/6 mice were purchased from Charles River Laboratories (Tokyo, Japan). *Exoc1*^*tm1a (EUCOMM)Hmgu*^ mice were obtained from the International Knockout Mouse Consortium and the International Mouse Phenotyping Consortium [48] and *Exoc1*^*tm1b (EUCOMM)Hmgu*^ (referred as *Exoc1*^*LacZ*^) and *Exoc1*^*tm1c (EUCOMM)Hmgu*^ (referred as *Exoc1*^*flox*^) mice were derived from *Exoc1*^*tm1a (EUCOMM)Hmgu*^ mice using the same methods as described in a previous report [17]. B6-*Ddx4*^*em1(CreERT2)Utr*^ mice (referred as *Ddx4*^*+/CreERT2*^) were generated as described were produced in our previous report [21]. We used B6-*Exoc3*^*em1(flox)Utr*^ (referred as *Exoc3*^*flox*^) and B6-*Exoc7*^*em1(flox)Utr*^ (referred as *Exoc7*^*flox*^) mice generated from our previous reports [49, 50]. The genetic background of all genetically modified or wild-type mice used in the experiments were C57BL/6 mice. Genotyping of the *Exoc1*^*flox*^ allele was formed in the same way as previously described [16].

### *Gdf9*^*Cre*^ mouse production through using zygote genome editing

The knock-in efficiency in cultured cells was increased using the fusion via fusing Cas9 with human GEMININ, which was not degraded only during the S-M phase when knock-in events occurred [51]. We applied this technology was applied to mouse embryo genome editing. Standard Cas9 was replaced with Cas9-mouse GEMININ in *pX330* (Addgene # 42230) [52] to yield *px330-mG*. This vector was deposited to RIKEN BRC (RDB14405). We selected a A sequence (5´-GTG GCC CCC ATG CTA ACG AC-3´) containing the termination codon of *Gdf9* was selected as the sgRNA target. We inserted this sequence was inserted into the *pX330-mG* plasmid and designated as *px330-mG-Gdf9* (RDB14408). A P2A-NLS cre-rabbit globin polyadenylation sequence was present between the 5´ and 3´ homology arms of the donor DNA and was, designated as *p-Gdf9-cre-KI* (RDB14409);: each genomic sequence from 1377 bp upstream to immediately before the *Gdf9* termination codon of *Gdf9* and the genome region from immediately before the termination codon to 1444 bp downstream of the termination codon was used as 5´-homology and 3´-homology arms, respectively. The DNA vectors were isolated using the FastGene Plasmid Mini kit (Nippon Genetics, Tokyo, Japan) and filtered using a MILLEX-GV 0.22 μm filter unit (Merck Millipore, Darmstadt, Germany) for microinjection.

Pregnant mare serum gonadotropin (5 units) and human chorionic gonadotropin (5 units) were intraperitoneally injected into female C57BL/6 J mice at a 48-h interval and mated with male C57BL/6 J mice. A mixture of *p-Gdf9-cre-KI* (10 ng/µl) and *px330-mG-Gdf9* (5 ng/µl) was injected into the pronuclei of 146 zygotes. Surviving injected zygotes were transferred into the oviducts of pseudo-pregnant ICR females and 35 new-borns were obtained.

To confirm the designed knock-in mutation, genomic DNA was purified from the tail with PI-200 (Kurabo Industries Ltd., Osaka, Japan) according to manufacturer’s protocol. Genomic PCR polymerase chain reaction was performed using KOD-Fx (Toyobo, Osaka, Japan). Primers (GDF9-G5F: 5´-CCT AGG GTT CAA ACT CAA GTC CTC AAG C-3´ and GDF9-G3R: 5´-TGT GAA GTC AGA AAG GAA AAA CCG AGT G -3´) were used to check for correct knock-in mutations. We found that a total of 10 founders carried the designed mutations. In addition, we checked the random integration of *pX330-mG* and *p-Gdf9-cre-KI* by was checked using polymerase chain reaction PCR with an ampicillin resistance gene-detecting primer (Amp detection F: 5´-TTG CCG GGA AGC TAG AGT AA-3´, and Amp detection: R: 5´-TTT GCC TTC CTG TTT TTG CT-3´).

### Haematoxylin and Eosin (H&E) staining

Paraffin sections (5 µm) were deparaffinized and fixed in 10 NM Mildform for 30 min. The sections were placed in Meyer’s haematoxylin solution (Fujifilm Wako Pure Chemical Co., Ltd., Osaka, Japan) for 15 min at 20–25 °C. The slices were then rinsed with water. The samples were placed in 1% eosin Y solution (Fujifilm Wako Pure Chemicals Co., Ltd., Osaka, Japan) for 5 min at 20–25 °C. The slices were dehydrated, permeabilised with ethanol and xylene, and sealed with EUKITT mounting medium for microscopy (Mikroskopische Gläser, O. Kindler GmbH, Bobingen, Germany). The H&E-stained samples were observed under an all-in-one fluorescence microscope BZ-X710 (KEYENCE, Osaka, Japan).

### Immunofluorescence, lectin, and X-gal staining

The frozen sections were dried using a hair dryer and washed twice for 10 min in phosphate-buffered saline (PBS). Permeabilizsation was performed through via incubation with 0.25% Triton X-100 in PBS solution for 30 min at 20–25 °C. The sections were washed twice with PBS for 10 min and blocked with blocking buffer (10% goat serum, 0.01% tween-20, and 0.1% bovine serum albumin in PBS) or Blocking One Histo (Nacalai Tesque Co., Ltd., Kyoto, Japana) for 60 min at 20–25 °C. The sections were incubated with primary antibody solution at 37 °C for 120 min, washed twice with PBS for 10 min, and then incubated with secondary antibody solution for 60 min at 20–25 °C while shielded from light. After washing twice with PBS for 10 min, the sections were stained with wheat germ agglutinin (WGA) CF 488 A or 640 R conjugate (Biotium, CaliforniaCA, United States) for 15 min at 20–25 °C. Nuclei were stained with 4’,6-diamidino-2-phenylindole (DAPI) for 5 min at 20–25 °C, washed with PBS for 5 min, and covered with prolong gold antifade reagent with DAPI (Thermo Fisher Scientific, MassachusettsMA, United States).

Paraffin sections were de-paraffinised and incubated with 0.25% Triton X-100 in PBS solution for 20 min at 20–25 °C for permeabilisation, washed twice with PBS for 10 min, and then immersed in Target Retrieval solution (Agilent Technologies, California CA, United States). The sections were washed twice with PBS for 10 min and autoclaved with Target Retrieval solution at 121 °C for 10 min. The sections were then washed twice with PBS for 10 min, and blocking was performed in the same way as similar to that for the frozen sections. Sections were incubated with primary antibody solution at 4 °C overnight or at 20–25 °C for 60 min. The sections were washed twice with PBS for 10 min and subjected to a secondary antibody incubation, WGA staining, nuclear staining, and inclusion using the same procedure as for the frozen sections. All the antibodies used, and their dilutions are shown in Supplementary Table 1. Each section was observed using an all-in-one fluorescence microscope BZ-X710 or confocal microscope Leica SP8 (Leica Microsystems GmbH, Wetzlar, Germany).

### Oocyte counting and follicle stage determination

Serial paraffin sections (5-µm thick) were stained with H&E. Four serial sections (20 µm) were selected from a total of the 12 sections (60 µm) because they contained the highest number of oocytes. However, the whole ovary sections of *Exoc1-D-*cKO-E and 10-week-old *Exoc1-D-*ctrl-E mice were counted due to the considerable difference in ovary size. Follicles in which oocytes were surrounded by a single layer of flat or cuboidal granulosa cells were counted as primordial or primary follicles, respectively. Oocytes surrounded by two layers of granulosa cells were counted as secondary follicles, and follicles larger than secondary follicles with cavities were counted as antral follicles.

### Oocyte diameter determination

Paraffin-embedded ovaries were sliced into 5 µm-thick serial sections and stained with H&E. For each condition, the oocytes in primordial or primary follicles were randomly selected from 12 (60 µm) serial sections of the ovary, and their diameters were measured using ImageJ software.

### Ovary transplantation

The recipient was a 19-week-old *R26*^*GRR*^ mouse generated in a previous study [19]. After administration of the three types of mixed anaesthetic agents [53], the dorsal skin was incised to expose the ovaries. A small hole was made in the ovarian capsule and a partial incision was made in the recipient ovary. To obtain donor ovaries, three3-week-old mice were treated with 10% pentobarbital in saline solution. The donor ovaries were inserted into the slit in the ovaries of the recipient ovaries, as described above. The donor mice were euthanised immediately after ovary collection. In the ovulation induction experiment, superovulation was induced in recipient mice nine weeks after transplantation through the via administration of pregnant mare serum gonadotropin PMSG and hCG human chorionic gonadotropin at 48-h intervals, and oocytes were collected from the oviducts. These oocytes were fertilised with wild-type mouse sperm using in vitro fertilisation, and green fluorescent protein (GFP) fluorescence was confirmed under using a fluorescent stereomicroscope. In the experiment with no ovulation induction, recipient mice were cardiac-perfused with PBS solution under anaesthesia nine weeks after transplantation and ovaries were collected. Ovaries containing donor grafts were subjected to H&E staining and fluorescence immunostaining with an anti-GFP antibody and oocyte counting, as previously described.

### Image analysis of immunostaining signals

Ovarian paraffin sections were sliced into 5-µm sections and fluorescently immunostained with WGA and c-KIT, or WGA, and the immunostaining signals were detected using a Leica SP8 confocal microscope (Leica Microsystems GmbH). WGA and GDF9 co-fluorescence immunostaining were performed on paraffin sections thinly sliced to 20 µm. The areas of oocytes or follicles were defined based on the WGA-stained images, and the intensity and area of each immunostaining signal within these areas were calculated using ImageJ software. The The intensity and area of each immunostaining signal in the WGA signal-positive area were calculated using ImageJ software.5 µm sectioning slides were co-immunostained with FOXO3a and MVH. MVH and DAPI, respectively, defined the areas of oocytes and nuclei, respectively. A Leica SP8 confocal microscope (Leica Microsystems GmbH) was used to detected fluorescent images., and ImageJ software calculated tThe intensity of the nucleus and cytoplasmic FOXO3a signal was calculated using ImageJ software.

### Administration of PTEN inhibitor administration

Following the methodology as described in a previous study [29], bpV (HOpic) (AdipoGen Life Sciences, Inc., California CA, United States) was injected intraperitoneally at 150 ng per gram of body weight into mice at 5, 18, and 42 days post-natall days.

### Tamoxifen administration

To delete *Exoc1* in oocytes of adult mice, each eight8-week-old mouse was injected with 100 µl of 20 mg/ml Tamoxifen (Sigma-–Aldrich, MO, USA) in corn oil, following the preparation protocols described previously [21]. The mice were injected on four consecutive days, three resting days without injection, and four consecutive days.

To delete *Exoc1* in oocytes of embryonic mice, Tamoxifen (Sigma-–Aldrich) was dissolved in peanut oil up to 75 mg/ml and shaken at 56 °C overnight. An oral gavage tube was inserted from the mouth to the stomach of pregnant mice. Each mouse was pumped once with 100 µl at E15.

### PCNA-positive granulosa cell counts in follicles

Ovarian paraffin sections of 5 µm were co-fluorescence-immunostained with FOXL2 and PCNA., and randomly selected primary follicles were observed using a fluorescence SP08 confocal microscope. The total number of granulosa cells and the number of PCNA-positive granulosa cells per primary follicle were calculated from FOXL2- and PCNA-stained images.

## Supplementary information

Supplementary Table 1

Supplementary Figures 1-11

## Data Availability

All data supporting the findings of this study are available within the paper and its Supplementary Figs. 1–11 and Supplementary Table 1. PCR genotyping primers sequence are provided in Methods and Materials.
