# Supplementary material for: Exocyst complex component 1 (Exoc1) loss in dormant oocyte disrupts c-KIT and growth differentiation factor (GDF9) subcellular localization and causes female infertility in mice
Source: Cell Death Discov. 2025 Jan 20;11:17. doi: 10.1038/s41420-025-02291-5 (PMC11747099; doi:10.1038/s41420-025-02291-5)
Supplement: Supplementary file 2 — Supplementary Figures 1-11 [file 41420_2025_2291_MOESM2_ESM.docx]

**
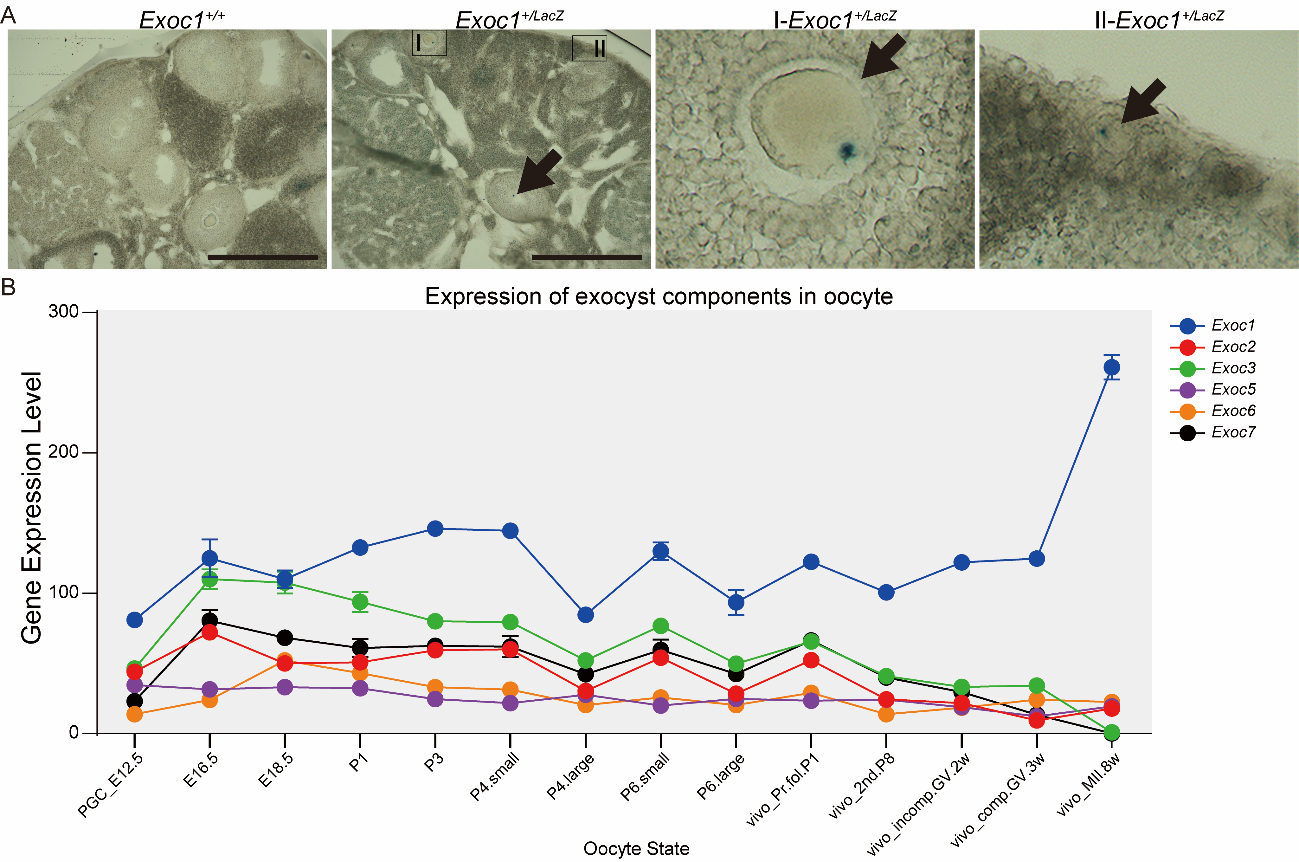
**

**Supplementary Figure 1**: ***Exoc1* expression in mouse oocytes.**

**A.** X-gal staining of ovaries from *Exoc1^+/LacZ^* mice to confirm *Exoc1* expression in mouse oocytes. LacZ signals were detected in immature and maturing oocytes. Arrow: oocytes. I- *Exoc1^+/LacZ^*: maturing oocyte. II- *Exoc1^+/LacZ^*: immature oocyte. Scale bar = 500 μm. **B.** Expression of exocyst complex in oocytes, confirmed using available RNA-Seq data (GSE143218). All members of the exocyst complex were expressed in various-staged oocytes.

**
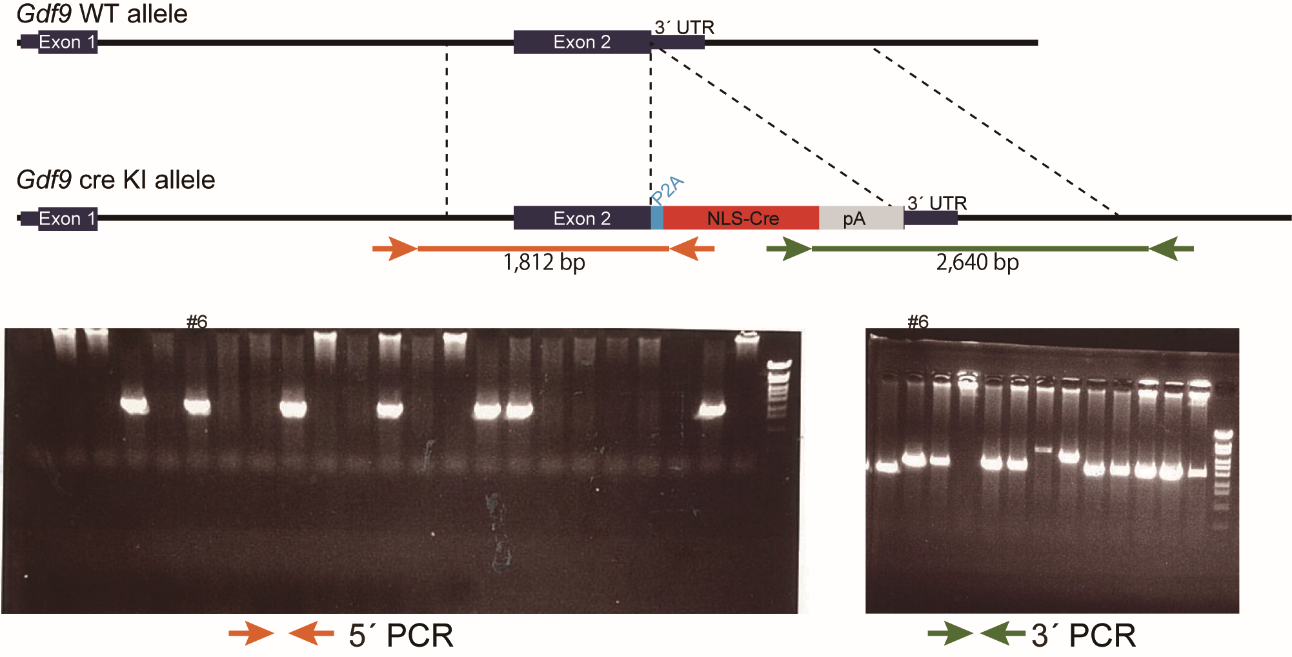
**

**Supplementary Figure 2: *Gdf9*-Cre knock-in mice generation.**

Strategy of generating *Gdf9*-Cre knock-in mice. To construct the knock-in allele, a P2A connected with NLS-Cre rabbit globin polyadenylation sequence was knocked-in immediately before the *Gdf9* stop codon. Knock-in alleles were confirmed by PCR genotyping. Mice #6 was selected as the founder mice of *Gdf9-*Cre strain.

**
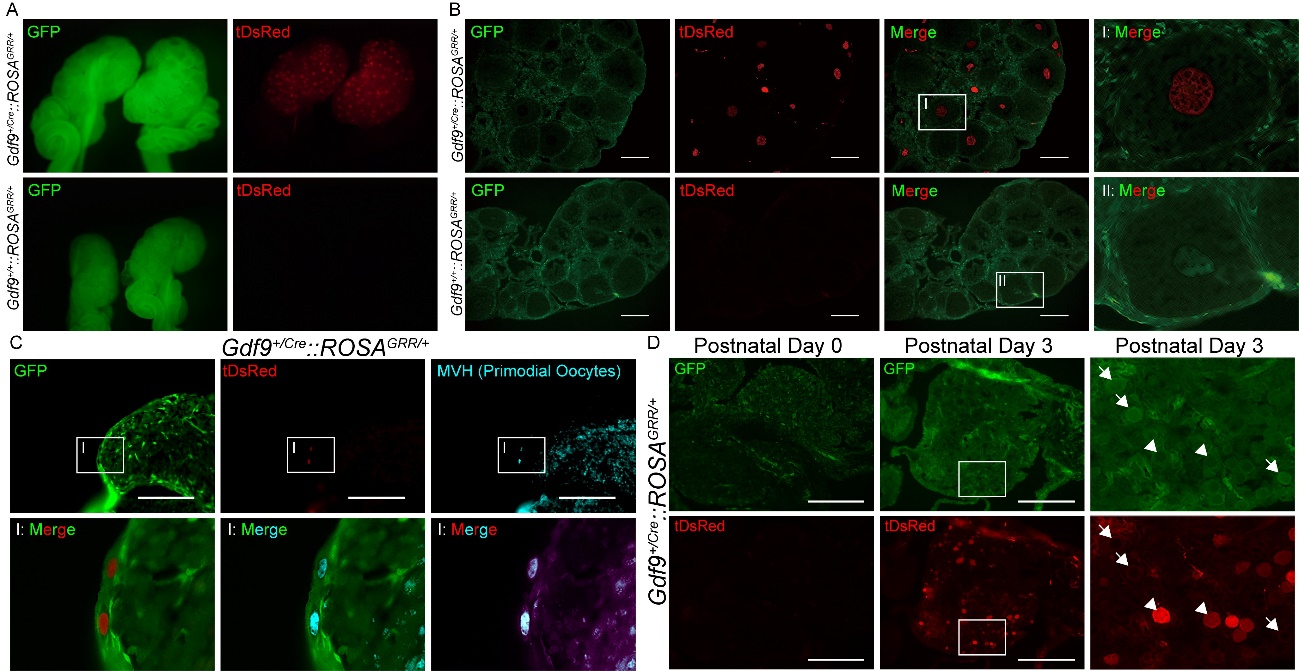
**

**Supplementary Figure 3: Cre recombination activity in *Gdf9*-Cre mice oocytes.**

*Gdf9^+/Cre^* mice were crossed with *Gt(ROSA)26Sor^tm1(CAG-EGFP/tDsRed)Utr^* referred to *ROSA^GRR^* mice carrying a green-to-red fluorescent-convertible Cre reporter. **A.** A tDsRed signal was observed in the ovaries of *Gdf9^+/Cre^;;ROSA^GRR/+^* mice using a fluorescence stereomicroscope. **B.** The tDsRed signal was present only in oocytes, and not in somatic cells. Scale bar = 50 μm. **C.** Cre recombination occurred from primordial oocytes. Scale bar = 200 μm. **D.** The timepoint when Cre recombination began was determined. On postnatal day 0 (P0), no tDsRed signal was detected. On P3, tDsRed-positive oocytes were seen in the *Gdf9^+/Cre^;;ROSA^GRR/+^* ovary. Some green fluorescent protein-positive oocytes remained present in this mice line. This confirmed that Cre recombination in *Gdf9*-Cre mice began from P3, and the oocytes were completely recombined at the adult stage. Arrowheads: oocytes. Scale bar = 200 μm.

**
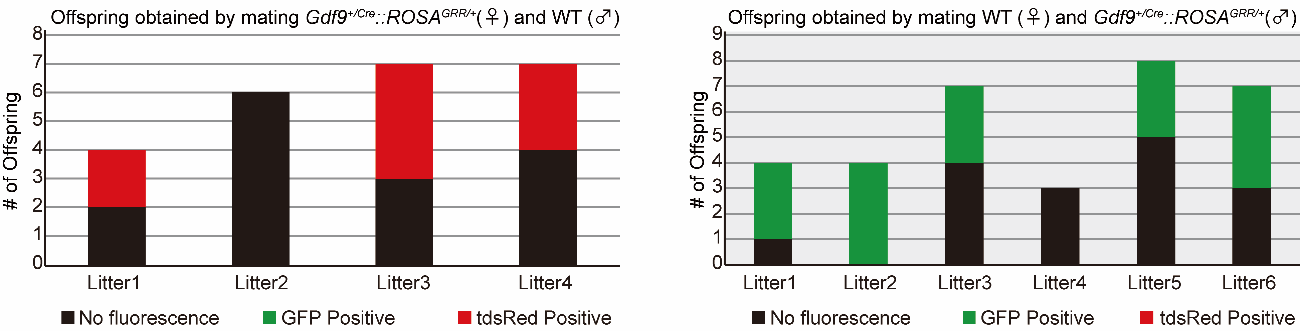
**

**Supplementary Figure 4: Cre recombination efficiency in *Gdf9*-Cre mice.**

To verify recombination in oocytes, offspring obtained from crossing *Gdf9^+/Cre^;;ROSA^GRR/+^* female mice and wild-type male mice were examined. Four litters were checked. No green fluorescent protein-positive offspring were observed, confirming that recombination occurred in all *Gdf9^+/Cre^* female mice oocytes. To verify recombination in sperms, six litters of offspring from *Gdf9^+/Cre^;;ROSA^GRR/+^* male crossed with wild-type female were examined. No tDsRed-positive offspring were observed, showing that Cre recombination did not occur in *Gdf9^+/Cre^* male mice.


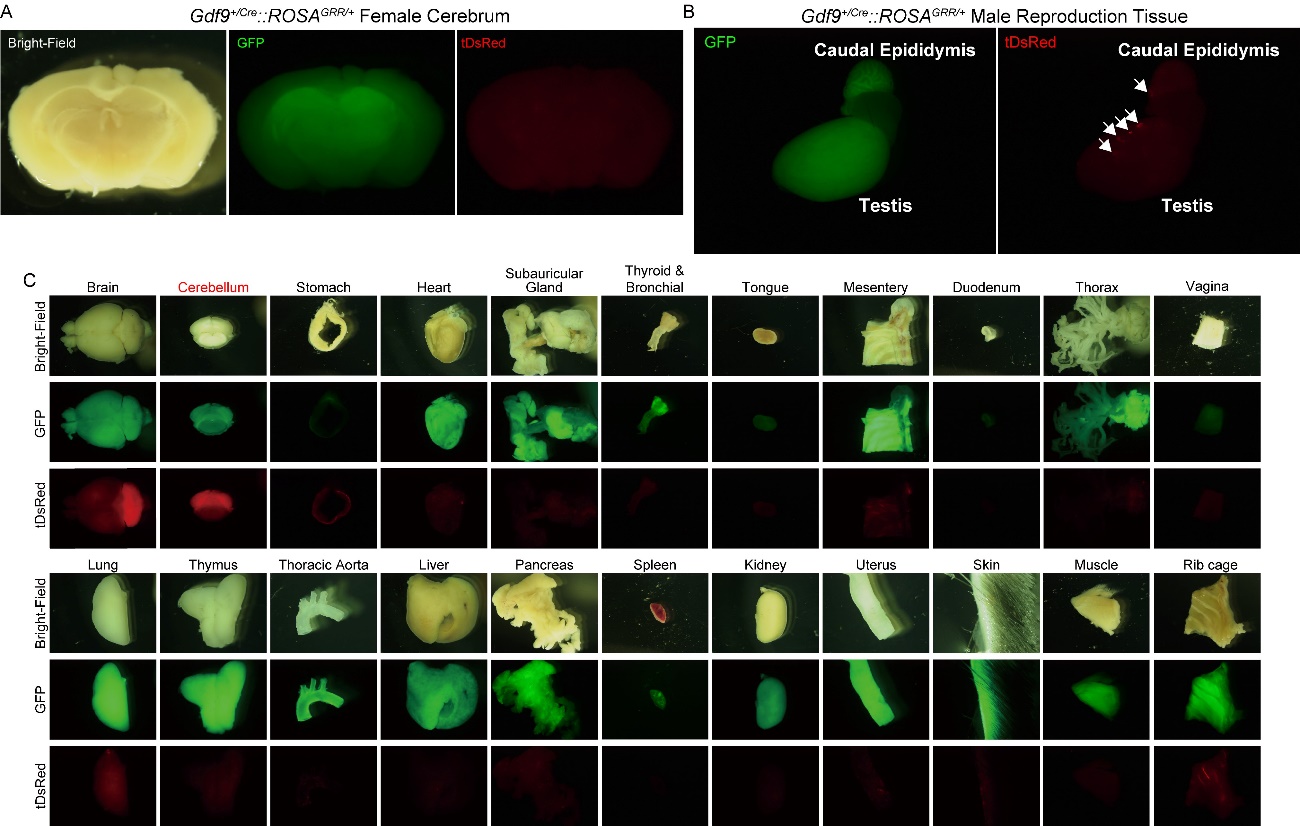


**Supplementary Figure 5: Cre recombination in various tissues in *Gdf9*-Cre mice.**

**A.** Recombination in *Gdf9^+/Cre^;;ROSA^GRR/+^* female cerebrum. No recombination was observed in the hypothalamus. **B.** Recombination in *Gdf9^+/Cre^;;ROSA^GRR/+^* male reproduction tissue. The tDsRed signals were found in only in few testis cells. Arrowheads: The tDsRed signal detected in testis. **C.** Cre recombination in other tissues. The tDsRed signal was found in the cerebellum. No tDsRed signals were detected in other tissues.
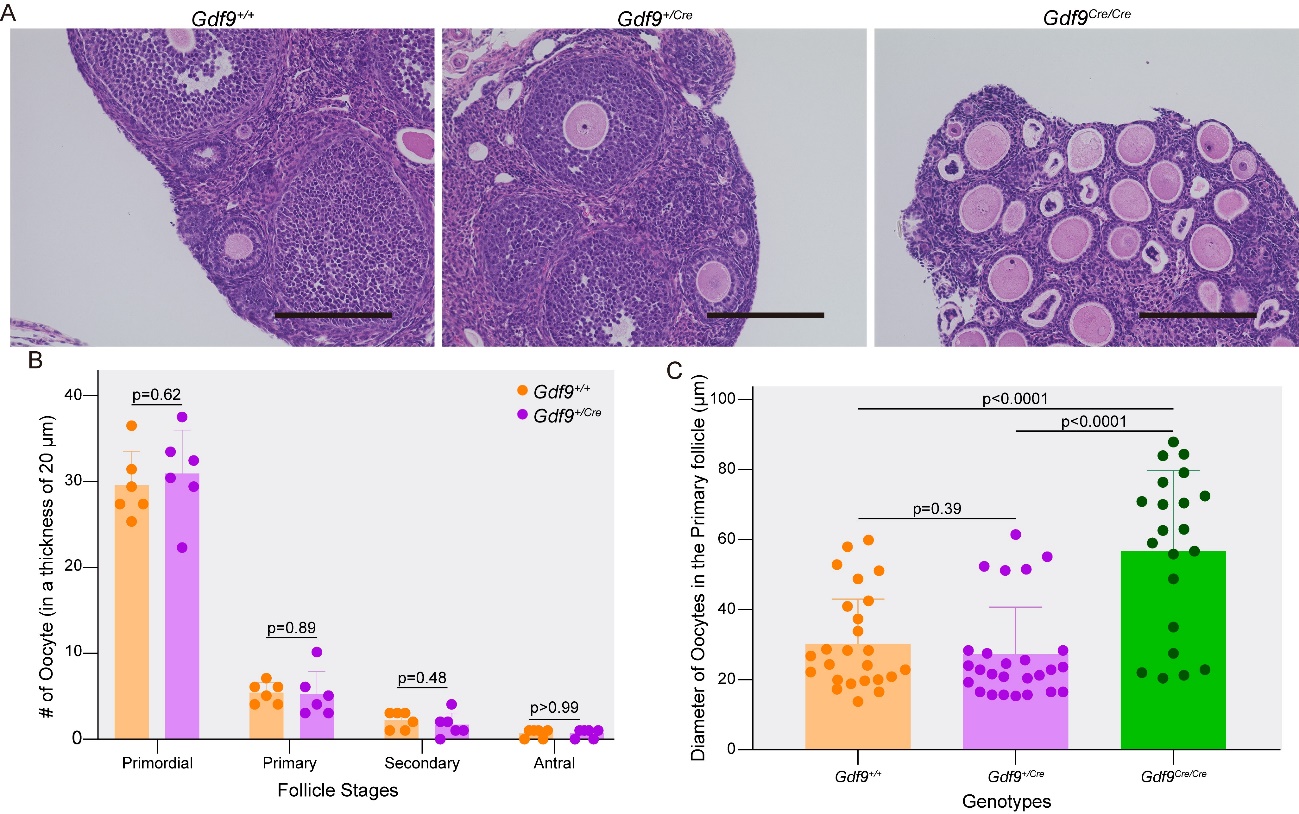


**Supplementary Figure 6: Phenotype of *Gdf9*-Cre female mice.**

**A.** Macroscopic Haematoxylin and Eosin staining images of the ovaries of each genotype mouse. Normal histological morphology was observed in heterozygous *Gdf9^+/Cre^* mice. Conversely, in *Gdf9^Cre/Cre^* mice, primary oocytes had increased diameters, and secondary oocytes or later stages of oocyte development were absent. Scale bar = 200 μm. **B.** Number of oocytes at each stage, including primordial, primary, secondary, and antral oocytes, was recorded. The number of oocytes at each stage was comparable between the wildtypes and *Gdf9^+/Cre^* mice (n = 5, Student’s *t*-test). **C.** The diameter of primary oocytes in *Gdf9^Cre/Cre^* mice. Homozygous *Gdf9^Cre/Cre^* mice showed a significant increase in oocyte diameter at the primary stage (n = 5, one-way analysis of variance), confirming that these oocytes failed to proceed to further developmental stages beyond the primary stage.


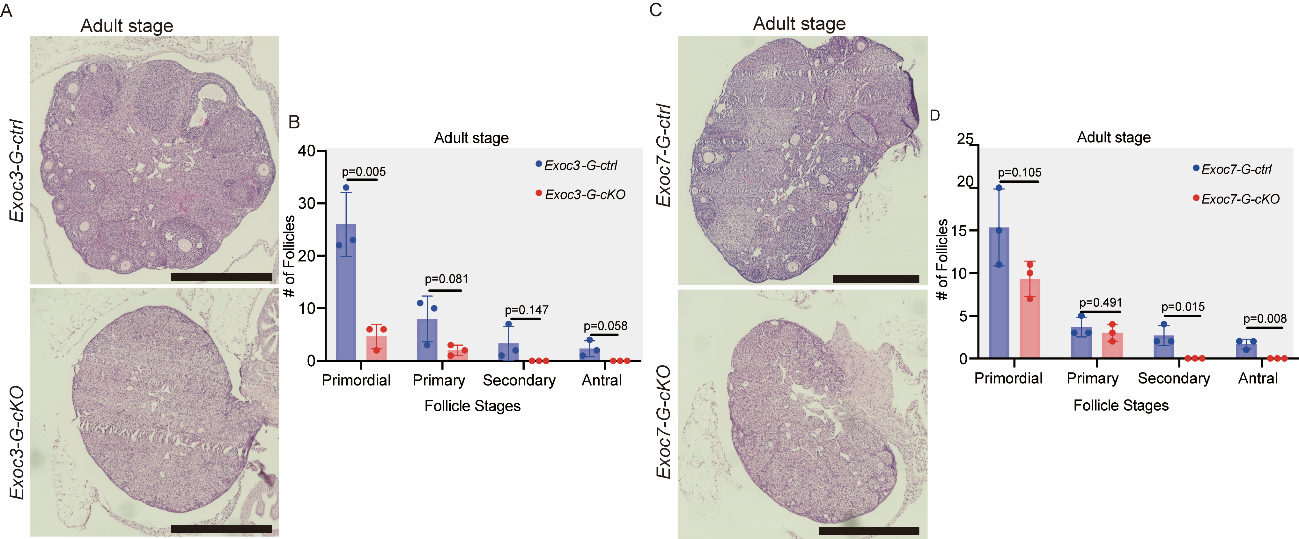


**Supplementary Fig 7**: **Phenotype *Exoc3* and *Exoc7* conditional knockout female mice.**

**A.** Representative Haematoxylin and Eosin images of adult *Exoc3-G-*cKO and **C.** adult *Exoc7-G-*cKO ovary. Scale bar = 500 µm. **B.** Oocyte count in ovaries in *Exoc3-G-*cKO and **D.** *Exoc7-G-*cKO mice. No secondary nor antral follicles were observed in both *Exoc3-G-*cKO and *Exoc7-G-*cKO mice at the adult stage. n = 3, Student’s *t*-test.


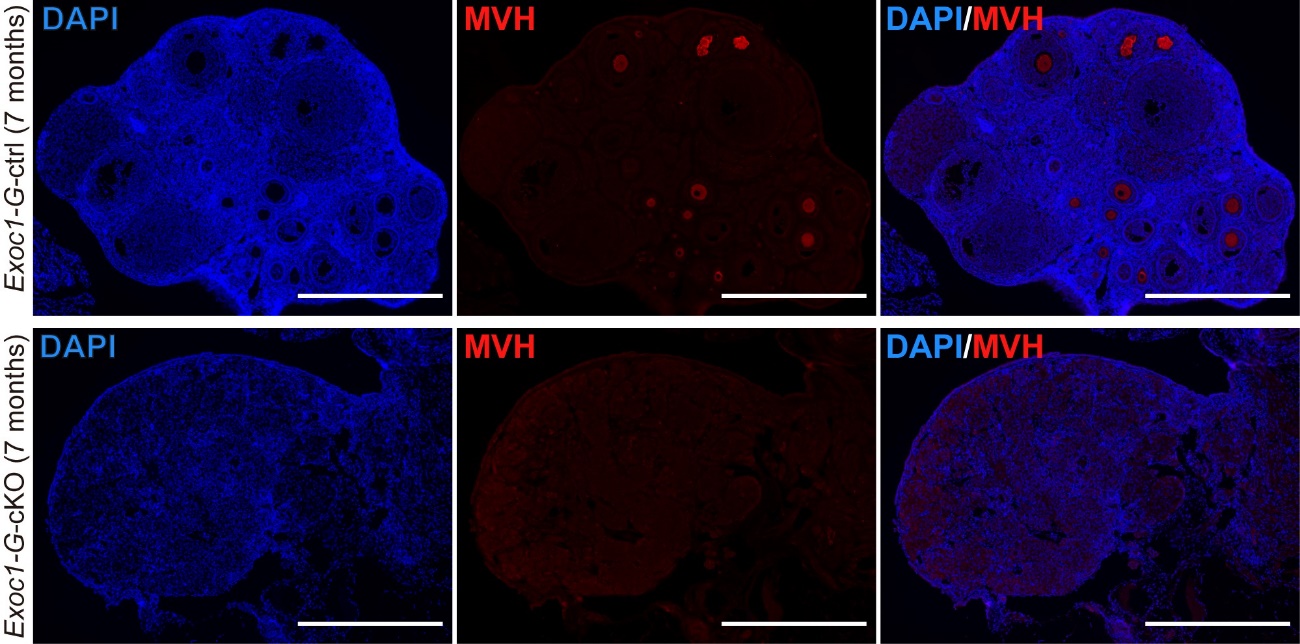


**Supplementary Figure 8: No follicles observed in aged *Exoc1-G-*cKO mice.**

To confirm the presence of oocytes, we performed immunofluorescence staining for MVH, a marker of oocytes from seven-month-old *Exoc1-G-*ctrl and *Exoc1-G-*cKO mice. No oocytes were found in the ovaries of aged *Exoc1-G-*cKO mice. Scale bar = 500 μm.


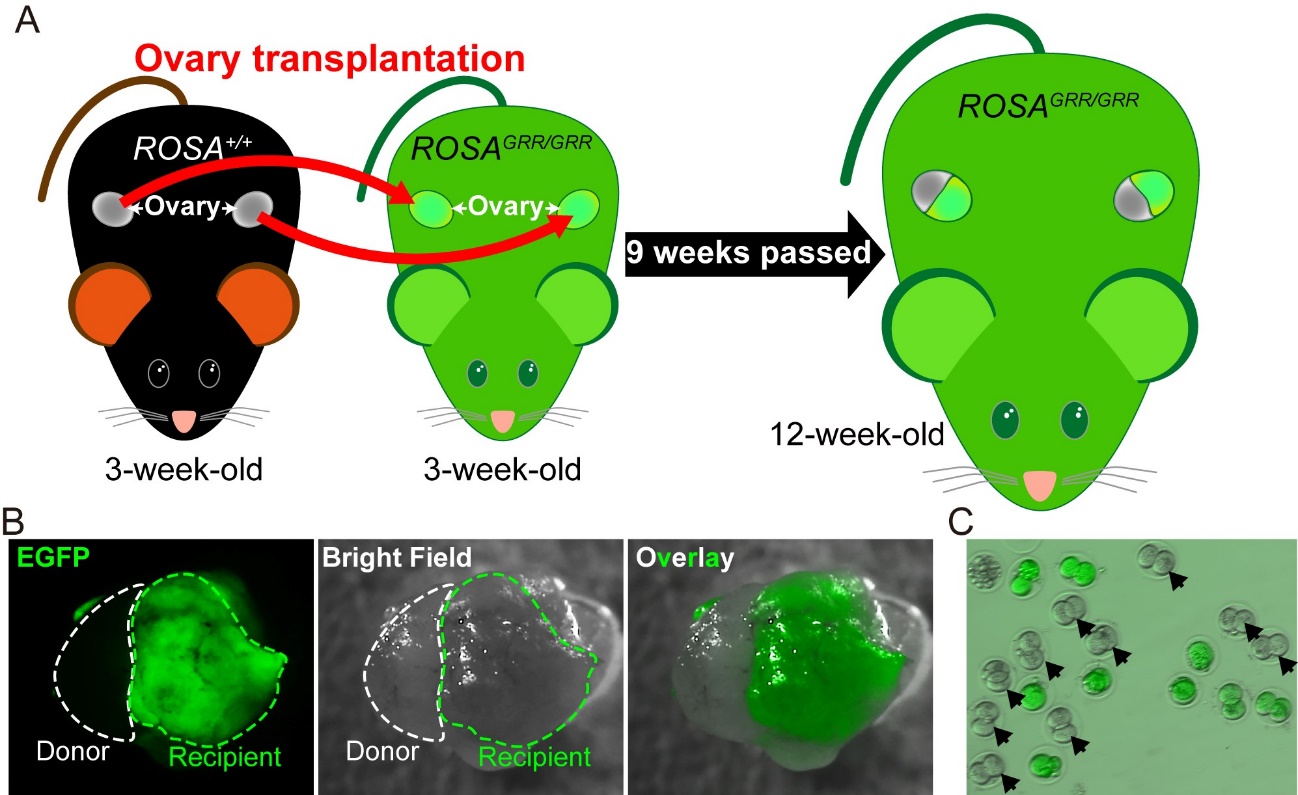


**Supplementary Figure 9: Ovarian transplantation of wildtype ovaries into *ROSA^GRR/GRR^* ovaries.**

**A.** The ovaries of three-week-old wildtype mice were transplanted into the ovaries of three-week-old *ROSA^GRR/GRR^* mice. The viability and ovulation capacity of the donor ovaries were determined nine weeks after the transplantation. **B.** Donor ovaries from wildtype mice were successfully implanted into recipient *ROSA^GRR/GRR^* mice ovaries. Then wildtype sperm were fertilized *in vitro* with oocytes from the recipient. **C.** The *in vitro* fertilization of wildtype sperm with oocytes collected from recipient mice oviducts. Some of the *in vitro* fertilized embryos at the 2-cell stage showed no fluorescent signals and are indicated by head arrows. This result clearly showed that ovarian transplantation at three weeks of age, when both the donor and recipient were not sexually mature, followed by waiting for the recipient to reach sexual maturity, resulted in the ovulation of fertilizable oocytes from the donor ovary.


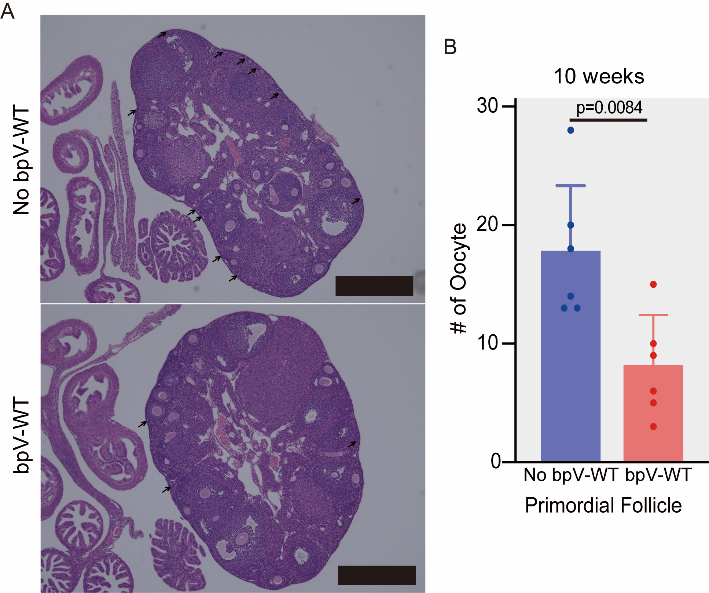


**Supplementary Figure 10: bpV injection to wildtype mice**

To test the inhibitory ability of bpV on PTEN to activate the oocyte, bpV was injected to wildtype mice. **A.** Representative ovary of 10-week-old wildtype mice injected without or with bpV. Scale bar = 500 µm. Arrows: primordial follicles. **B.** The number of primordial follicles of each group. n = 6, Student’s *t*-test. The number of primordial follicles in the wildtype mice injected with bpV was significantly lower than that in the group without bpV injection, which indicates the inhibitory functions of bpV on PTEN during oocyte activation.


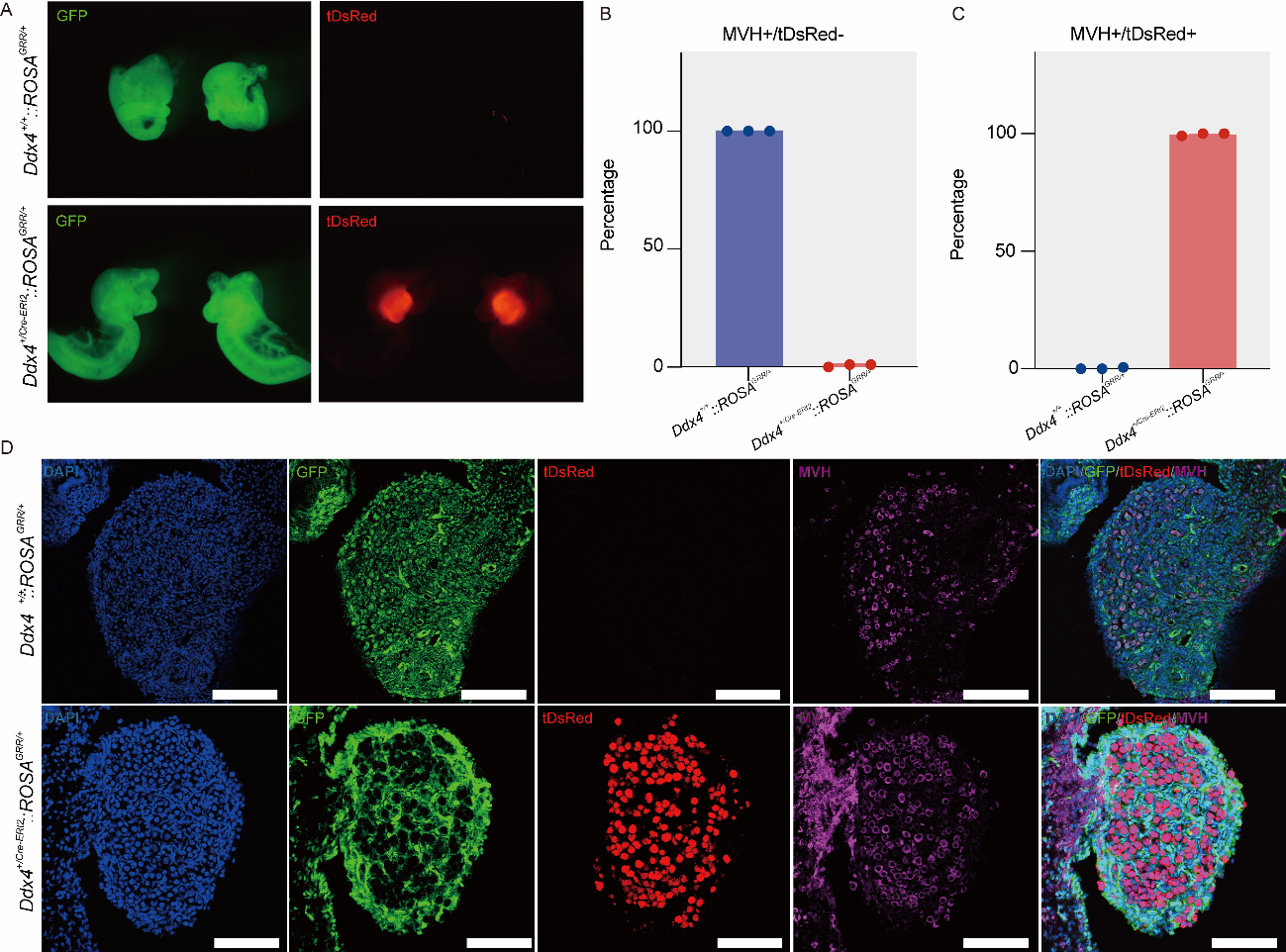


**Supplementary Figure 11: Efficiency of Cre-recombination induced by tamoxifen during pregnancy**

**A.** Representative ovary collected at postnatal day 0 (P0) of *Ddx4^+/+^*::*ROSA^GRR/+^* and *Ddx4^+/CreERT2^*::*ROSA^GRR/+^* female mice. **B.** Bar chart of the percentage of MVH+/tDsRed- and **C.** MVH+/tDsRed+ of both *Ddx4^+/+^*;;*ROSA^GRR/+^* and *Ddx4^+/CreERT2^*;;*ROSA^GRR/+^* groups. Only the *Ddx4^+/CreERT2^*;;*ROSA^GRR/+^* ovaries show the conversion from green to red. **D.** Representative microscopic images of ovaries in *Ddx4^+/+^*;;*ROSA^GRR/+^* and *Ddx4^+/CreERT2^*;;*ROSA^GRR/+^* female mice. Scale bar = 500
